# Menstrual variations of sleep–wake rhythms in healthy women

**DOI:** 10.1007/s41105-024-00543-y

**Published:** 2024-07-11

**Authors:** Tomoko Namie, Tsugumi Kotaka, Kazuto Watanabe, Nana N. Takasu, Wataru Nakamura, Takahiro J. Nakamura

**Affiliations:** 1 Laboratory of Animal Physiology, School of Agriculture, Meiji University, 1-1-1 Higashimita, Tama-Ku, Kawasaki, Kanagawa 214-8571 Japan; 2 Department of Oral-Chrono Physiology, Graduate School of Biomedical Sciences, Nagasaki University, Nagasaki, 1-7-1 Sakamoto, Nagasaki, Nagasaki 852-8588 Japan

**Keywords:** Basal body temperature, Quasi-peak value, Sleep midpoint, Sleep duration, Social jetlag

## Abstract

The ovarian steroid hormones, estrogen and progesterone, the levels of which fluctuate dynamically with the estrous cycle, alter circadian behavioral rhythms in mammals. However, it remains unclear whether the sleep–wake rhythm fluctuates with the menstrual cycle in humans. To ascertain the relationship between the menstrual cycle and sleep–wake rhythms, we evaluated the objective and long-term sleep–wake rhythms of ten healthy women using a recently developed wearable device. The results showed a strong negative correlation between the sleep midpoint and the quasi-peak value (an indicator of rhythm robustness), and a positive correlation between the length of the menstrual cycle (days) and social jetlag (hours). These results suggest that healthy women with late sleeping habits have a disturbed sleep–wake rhythm and that irregular habits prolong the menstrual cycle. The sleep midpoint and quasi-peak values showed variations during the menstrual cycle. The quasi-peak values in the follicular phase were significantly higher than those in the menstrual and luteal phases. In rodents, the phase of locomotor activity rhythm advances, and activity increases at night during proestrus. The increase in quasi-peak values during the follicular phase, when estrogen is relatively high, may be due to the increased activity caused by estrogen. These results suggest that ovarian steroid hormones influence sleep–wake rhythms in women. Verifying the results of this study under various conditions is necessary; however, accurately predicting the day of ovulation using only the acquisition of sleep–wake rhythms with wearable devices will be possible.

## Introduction

Circadian rhythms are approximately 24-h oscillations in behavior and physiology that are internally generated and function to anticipate the environmental changes associated with the solar day. In mammals, circadian behavioral rhythms are altered by the ovarian steroid hormones estrogen and progesterone, which fluctuate dynamically with the estrous (referred to as menstrual in humans) cycle [1]. In the human menstrual cycle, estrogen levels increase in the follicular phase before ovulation, and progesterone levels increase in the luteal phase after ovulation. The average menstrual cycle lasts approximately 28 days; in rodents, the estrous cycle rotates within 4–5 days, with similar hormonal fluctuations. Estrogen levels increase through proestrus, while progesterone levels increase from proestrus to estrus [2–4]. Circadian activity rhythms change significantly during the estrous cycle in rodents [5, 6]. Female rats show phase advances in locomotor activity, and higher total activity, during proestrus and estrus than during diestrus [6]. Estrogen alters circadian rhythms, as implants shorten the period of locomotor activity in female rats and hamsters, while increasing amplitude and activity time [7, 8]. Interestingly, these estrogenic effects are alleviated in the presence of progesterone, suggesting that complex regulation of the hormonal milieu across the estrous cycle is necessary to bring about the observed behavioral effects [8].

Since humans are highly social animals, it is believed that alterations in activity rhythms associated with the menstrual cycle do not appear in real life as they do in rodents. However, in rare cases, alterations in sleep–wake rhythms, with sleep disturbances, have been reported [9, 10]. Sleep structure changes with the stage of the menstrual cycle in healthy women; for example, early onset of REM sleep occurs in the luteal phase, and arousal during sleep increases in the late luteal phase [11, 12]. Nocturnal slow-wave sleep does not change with the menstrual cycle [13]. However, examining the 24-h sleep structure after sleep deprivation reveals an increase in daytime slow-wave sleep during the luteal phase [14]. Thus, the effect of the menstrual cycle on sleep–wake rhythm in humans is poorly understood.

Based on previous reports, we hypothesized that the sleep–wake rhythm in healthy women is affected by the hormonal fluctuations of the menstrual cycle. This study aimed to ascertain the relationship between menstrual cycle stages and sleep–wake rhythms by obtaining objective data on long-term sleep–wake rhythms in healthy women using recently developed wearable technology [15–17]. If the relationship can be elucidated and its regularity determined, a wearable device, such as a smartwatch, could be used to identify the stages of the menstrual cycle and the day of ovulation. This information would be useful for family planning, infertility treatment, and management of mental and physical disorders, such as premenstrual syndrome and premenstrual dysphoric disorder.

## Materials and methods

### Participants

Participants were recruited from Meiji University (Kanagawa, Japan). The recruitment period was from March 1, 2021 to April 15, 2021, with data acquisition from May 1, 2021 to April 30, 2022. We included ten healthy women with regular menstrual cycles who were able to measure their sleep–wake rhythm and basal body temperature (BBT) and complete a self-administered questionnaire. The participants included four healthy women taking low-dose pills for menstrual pain. Although the menstrual cycle in these four women was shorter than that of the other six participants (*P* < 0.05, Student’s t-test), other parameters were indistinguishable between these two groups. Therefore, data from all the participants were analyzed.

### Procedures

Before participating in the study, participants completed the self-administered Pittsburgh Sleep Quality Index (PSQI) questionnaire, and the Munich Chronotype Questionnaire (MCTQ). The items of the self-administered questionnaire included age and regular medication. Before the intervention, participants were given a smartwatch (Apple Watch Series 3; Apple, Cupertino, CA, USA), a clinical thermometer (TDK, Tokyo, Japan) for BBT recording, and ovulation day prediction test drugs (Do-Test® LH II, ROHTO Pharmaceutical Co., Ltd., Osaka, Japan) to identify the day of ovulation.

#### BBT

The participants measured their BBT every morning using the clinical thermometer and recorded it on a smartphone application linked to the thermometer; Luna Luna (MTI Ltd., Tokyo, Japan) [18].

### Sleep parameters

The participants wore the smartwatch as much as possible during their daily activities, except when bathing; it was linked to a sleep-recording application (AutoSleep, Tantsissa, Australia) to measure sleep duration and depth. The participants regularly checked and corrected the data in the application and adjusted it so that it was as accurate as possible. Non-nighttime sleep, such as a nap, was also recorded and treated as one sleep section if no consecutive awakenings occurred for 120 min.

Sleep duration and sleep midpoint were defined as the interval between falling asleep and waking and the median of the time between falling asleep and waking, respectively. The acquired sleep–wake data were expressed as a number from 0 to 5 every 15 min according to the depth of sleep (0, awake; 3, shallow sleep; 5, deep sleep) and read using ClockLab software 33.(Actimetrics, Wilmette, IL, USA). Chi-square periodogram analysis was used to obtain the maximum quasi-peak (QP) value at approximately 24 h.

### Menstruation and ovulation day

The day of menstrual flow was recorded as the menstrual period, according to the participants’ self-reports. The day of ovulation was identified using an ovulation day prediction test (Do-Test LH II). The day after the detection of the luteinizing hormone (LH) surge was the day of ovulation. Although ovulation does not usually occur in low-dose pill users because of the effect of the pill, the Ogino theory [19], which states that ovulation occurs 12–16 days before the expected start of menstruation, was adopted. Therefore, 14 days before the start of menstruation was set as the provisional ovulation day for the pill users.

**Fig. 1:**
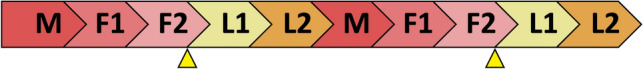
Five phases of the menstrual cycle. The yellow triangles represent the days of ovulation. The period from the start to the end of menstruation is defined as the menstrual phase, the period from the day after the end of menstruation to the day before ovulation is defined as the follicular phase, and the period from ovulation to the day before menstruation is defined as the luteal phase. The follicular and luteal phases are divided into stages I and II in the front and back halves, respectively, and the menstrual cycle is divided into five stages. *M* menstrual phase, *F1* follicular phase I, *F2* follicular phase II; *L1* luteal phase I, *L2* luteal phase II

The menstrual cycle stages were defined as the follicular phase from the day after the end of menstruation to the day before ovulation day, and the luteal phase from ovulation day to the day before the start of menstruation. The follicular and luteal phases were divided into stages I and II in the front and back halves, respectively, and the menstrual cycle was divided into five stages: the menstrual phase, follicular phase I, follicular phase II, luteal phase I, and luteal phase II (Fig. 1). If the number of days was odd when bisecting the follicular and luteal phases, the number of days in follicular/luteal phase II was 1 day more than the number of days in follicular/luteal phase I.

### Statistical analysis

For BBT, sleep duration, and sleep midpoint, the average value for 365 days was calculated for each participant to eliminate individual differences in the analysis of each stage of the menstrual cycle, and the variation from the average value (deviation) was used for comparison. Fluctuations in the QP value depend on the duration of the days being analyzed; therefore, the QP value for a given period was obtained and then divided by the period to calculate the value.

Statistical analyses were performed using the SPSS software (version 24.0; IBM Corp., Armonk, NY, USA). Correlations between the continuous variables of sleep duration, sleep midpoint, menstrual cycle length, PSQI score, and social jetlag (SJL) time from the MCTQ were evaluated using Pearson’s correlation coefficient. One-way analysis of variance (ANOVA) with Tukey’s post hoc test was used to compare the fluctuations in BBT, sleep duration, sleep midpoint, and QP value according to the menstrual cycle stage. The Student’s t-test was used to compare the two groups. All results are presented as means ± standard deviations and were considered significant at *P* < 0.05.

## Results

### Participants’ characteristics

Among the ten participants, four were low-dose pill users, and nine were university students at the beginning of the study. The mean age was 22.1 years, and the average length of the menstrual cycle during the experiment was 30.86 days. Participants’ mean sleep duration and sleep midpoint, and mean PSQI global score (PSQIG) and SJL, obtained from the questionnaire and MCTQ administered at the beginning of the experiment, respectively, are presented in Table 1. The mean QP value for a year, calculated using a chi-square periodogram, was 280,476.06.

**Table 1:** Participants’ characteristics

	Mean	Standard deviation
Age (years)	22.10	1.29
Menstrual cycle (days)	30.86	3.62
Sleep duration (min)	410	109
Sleep midpoint (local time, min)	5:21	79
PSQIG score	4.64	1.33
SJL (h)	0.77	0.50
QP value	280476.06	42772.93
BBT amplitude	0.26	0.09

*SD* standard deviation, *PSQIG* pittsburgh sleep quality index global score

*SJL* social jetlag, *QP* quasi-peak, *BBT amplitude* amplitude of the basal body temperature

When the correlations between the sleep duration, sleep midpoint, length of menstrual cycle, PSQIG, SJL, and QP values were evaluated (Table 2), no correlations were found between most variables. However, a strong negative correlation (r =  − 0.867, *P* = 1.15 × 10^−3^, Pearson’s correlation coefficient) was observed between the sleep midpoint and QP value and a positive correlation was observed between the menstrual cycle days and SJL (r = 0.638, *P* = 4.71 × 10^−2^, Pearson’s correlation coefficient). Furthermore, a positive correlation was found between the sleep midpoint and the PSQIG (r = 0.619), although the difference was not statistically significant.

**Table 2:** Pearson correlation coefficients and *P*-values for sleep duration, sleep midpoint, menstrual cycle days, PSQIG score, social jetlag, quasi-peak values, and amplitude of basal body temperature

	Sleep duration	Sleep midpoint	Menstrual cycle	PSQIG	SJL	QP value	BBT amplitude
Sleep duration	1.000						
Sleep midpoint	0.062 (NS)	1.000					
Menstrual cycle	0.175 (NS)	− 0.234 (NS)	1.000				
PSQIG	− 0.026 (NS)	0.619 (NS)	− 0.085 (NS)	1.000			
SJL	0.158 (NS)	0.187 (NS)	0.638*	0.166 (NS)	1.000		
QP value	− 0.138 (NS)	− 0.867**	0.189 (NS)	− 0.339 (NS)	− 0.286 (NS)	1.000	
BBT amplitude	− 0.098 (NS)	− 0.212 (NS)	0.730*	-0.073 (NS)	0.713*	0.247 (NS)	1.000

*NS* not significant,*PSQIG* pittsburgh sleep quality index global score, *SJL* social jetlag, *QP* quasi-peak, *BBT amplitude* amplitude of the basal body temperature. * P < 0.05; ** *P* < 0.01

### Menstrual variations in BBT

**Fig. 2:**
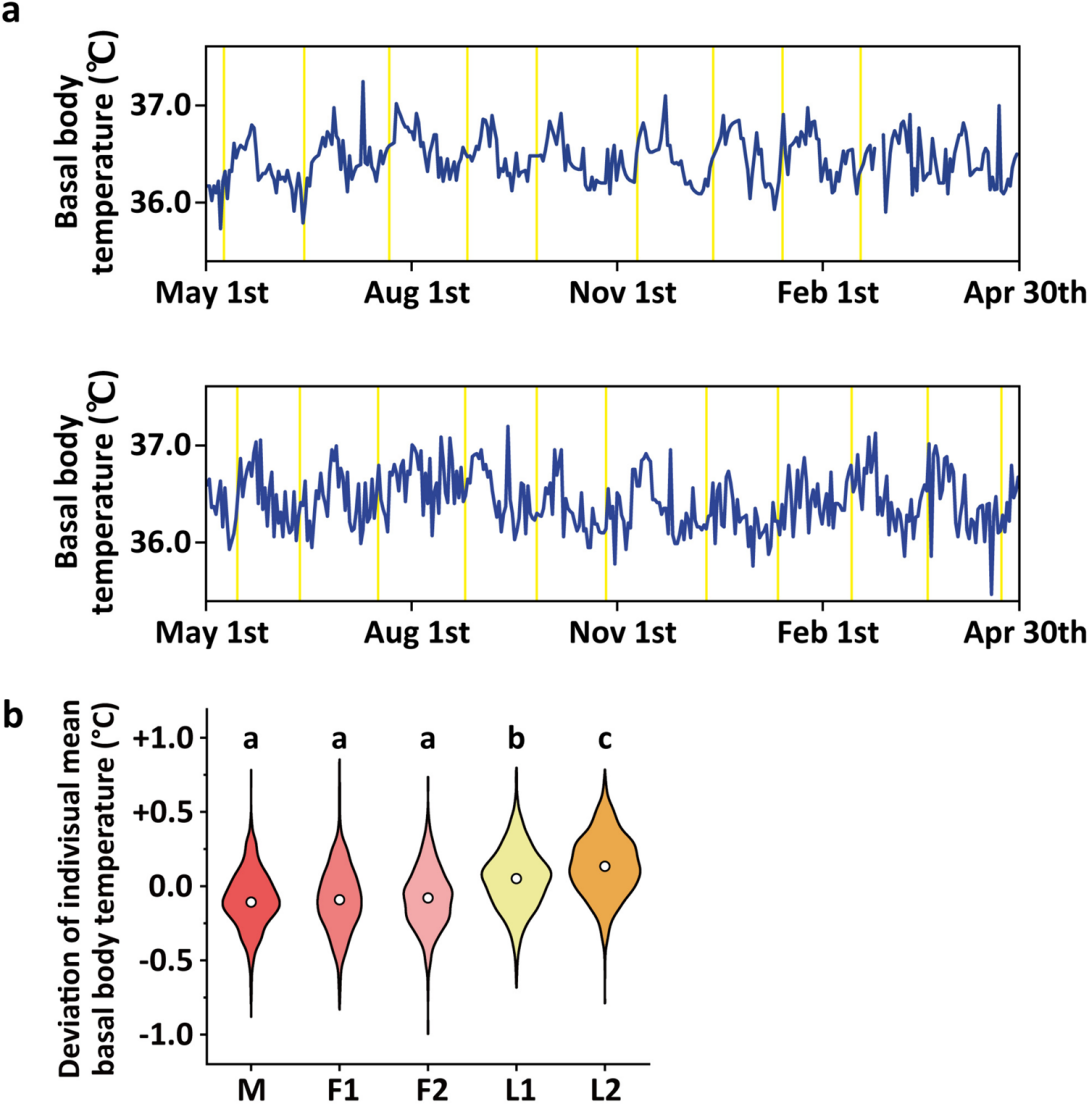
Menstrual variations in basal body temperature. **a** Representative graphs showing basal body temperature fluctuations over a year. The purple lines represent the basal body temperature, and the yellow lines represent the day of ovulation. **b** Violin plots showing the deviation of the average basal body temperature of each participant at each phase of the menstrual cycle. The white circles represent the mean values. *M* menstrual phase, *F1* follicular phase I, *F2* follicular phase II, *L1* luteal phase I, *L2* luteal phase II. Differing letters (a, b, and c) between groups indicate significant differences (*P* < 0.001, Tukey’s test)

Figure 2a shows representative graphs depicting BBT fluctuations over a year. Large individual differences in BBT were found; however, a biphasic pattern of low- and high-temperature phases was observed in all participants. Classifying the BBT into five menstrual stages (Fig. 1), and comparing the deviation from the average BBT of each participant yielded the following observations: − 0.09 ± 0.19 °C in the menstrual phase, − 0.09 ± 0.22 °C in follicular phase I, − 0.08 ± 0.20 °C in follicular phase II, + 0.05 ± 0.21 °C in luteal phase I, and + 0.14 ± 0.21 °C in luteal phase II, with significant stage-specific variation (*P* < 0.001, one-way ANOVA, *F* (4,3405) = 175.943). In a stage-by-stage comparison, the BBT in luteal phases I and II were significantly higher than those in the menstrual phase and follicular phases I and II (*P* < 0.001, Tukey’s test). BBT in luteal phase II was significantly higher than those in luteal phase I (*P* < 0.001, Tukey’s test; Fig. 2b). Additionally, the maximum and minimum values were extracted from the average BBT at each menstrual stage to ascertain BBT amplitude during each individual's menstrual cycle (Table 1). The BBT amplitude positively correlated with the length of the menstrual cycle and SJL (r = 0.730, *P* < 0.05 for BBT amplitude and length of menstrual cycle; r = 0.713, *P* < 0.05 for BBT amplitude and SJL; Pearson’s correlation coefficient, Table 2).

### Menstrual variations in sleep–wake rhythm

**Fig. 3:**
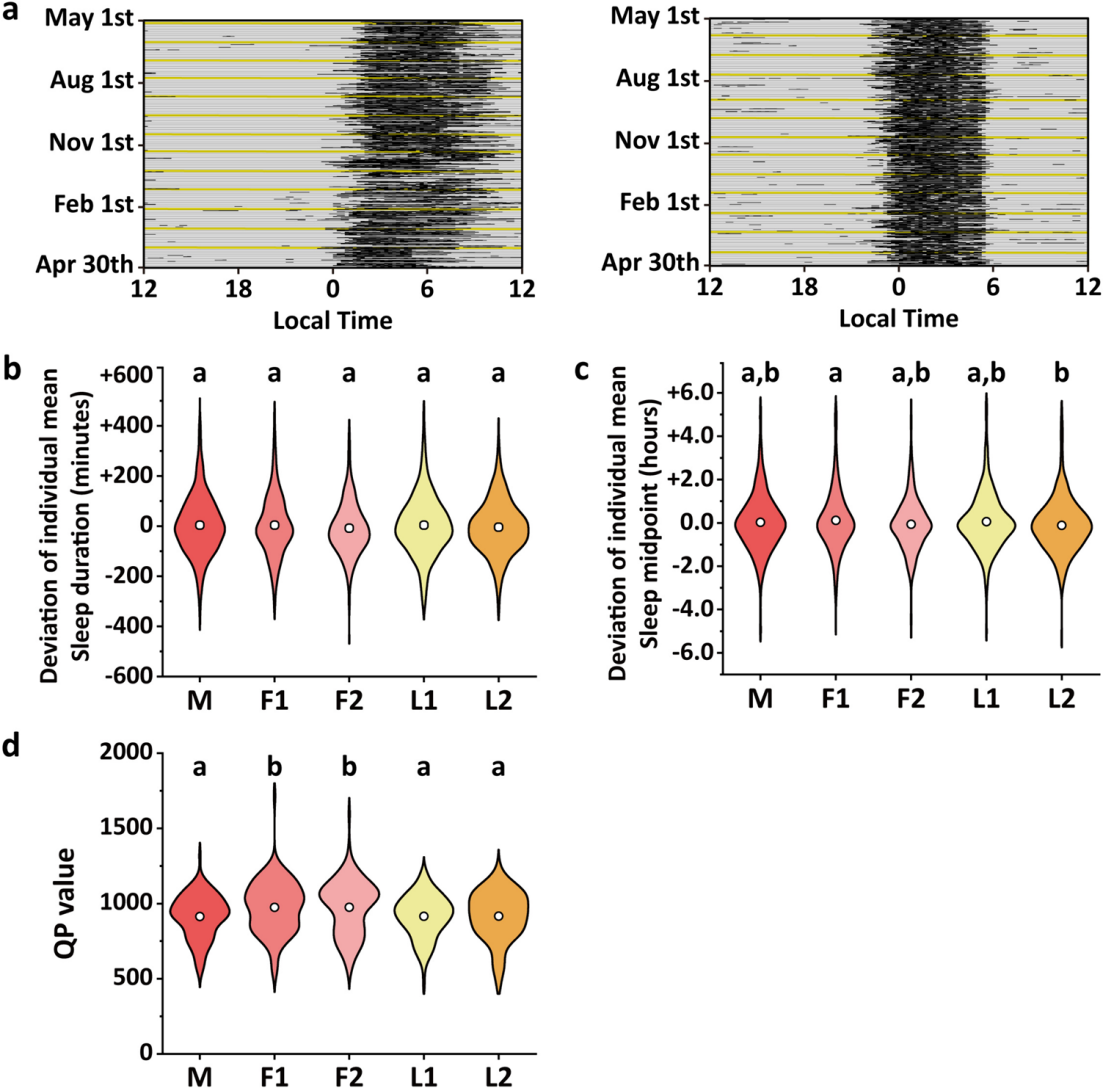
Menstrual variations in sleep–wake rhythm. **a** Representative graphs showing basal body temperature fluctuations over a year. The left panel represents an example of high sleep–wake rhythm variability and low QP value, and the right panel represents an example of low sleep–wake rhythm variability and high QP value. The vertical axis shows the date, and the horizontal axis shows the time. The black bars represent the sleep state, and the white bars represent the waking state. The density of the black bars indicates the depth of sleep. The yellow line represents the day of ovulation. **b**, **c** Violin plots showing the deviation of the average (**b**) sleep duration and (**c**) sleep midpoint of each participant at each phase of the menstrual cycle. **d** Violin plots showing QP values at each phase of the menstrual cycle. The white circles represent the mean values. *M* menstrual phase, *F1* follicular phase I, *F2* follicular phase II, *L1* luteal phase I, *L2* luteal phase II, *QP* quasi-peak. Differing letters (a and b) between groups indicate significant differences (*P* < 0.05, Tukey’s test)

Figure 3a depicts actograms representing the sleep–wake rhythm over a year. A large overall variation in the waking and sleeping times of the participants was observed. For sleep duration and midpoint, the deviation from the mean was determined for each participant. Sleep duration, sleep midpoint, and QP values were classified into five menstrual stages and compared.

Sleep duration was + 4 ± 116 min in the menstrual phase, + 4 ± 117 min in follicular phase I, − 8 ± 103 min in follicular phase II, + 4 ± 117 min in luteal phase I, and − 4 ± 104 min in luteal phase II, with no significant differences due to variations in the menstrual phase (*P* > 0.05, one-way ANOVA,* F* (4,3509) = 1.965; Fig. 3b).

The sleep midpoint was + 0.03 ± 1.24 h in the menstrual phase, + 0.12 ± 1.34 h in follicular phase I, − 0.06 + 1.28 h in follicular phase II, + 0.06 ± 1.35 h in luteal phase I, and − 0.11 ± 1.53 h in luteal phase II. Thus, the sleep midpoint showed significant variation by stage (*P* < 0.001, one-way ANOVA,* F* (4,3509) = 3.409). Based on the menstrual phase, the sleep midpoint was advanced during follicular phase I, delayed during follicular phase II, advanced during luteal phase I, and delayed during luteal phase II. Statistically, the sleep midpoint in follicular phase I was significantly earlier (*P* < 0.01, Tukey’s test) than that in luteal phase II (Fig. 3c).

The QP values were 914.43 ± 144.00 in the menstrual phase, 975.51 ± 173.34 in follicular phase I, 976.04 ± 181.04 in follicular phase II, 915.93 ± 141.40 in luteal phase I, and 916.96 ± 163.55 in luteal phase II, indicating significant stage-specific variation (*P* < 0.001, one-way ANOVA,* F* (4,590) = 4.939). The QP values in follicular phases I and II were significantly higher than those in the menstrual phase and luteal phases I and II (*P* < 0.05, Tukey’s test; Fig. 3d).

## Discussion

This study demonstrates that the sleep–wake rhythm in healthy women changes with the menstrual cycle stage. We demonstrated this by obtaining objective data on long-term sleep–wake rhythms with a wearable device. The gold standard for sleep analysis is polysomnography, which measures the electroencephalogram during sleep and can simultaneously record the electroencephalogram, respiration, leg movements, eye movements, electrocardiogram, and oxygen saturation to identify sleep stages in detail [20]. Although polysomnography is effective for testing sleep disorders in hospitals, it is complex and cannot be performed at home. Therefore, sleep diaries have been used as an alternative. In a sleep diary, individuals record their daily sleeping time, including naps, enabling long-term understanding of their sleep patterns. However, because this is subjective, an individual’s perception and the actual sleep state are often at variance [21]. Several devices and mobile applications have recently emerged that allow people sleep measurement inexpensively at home. Digital phenotyping using wearable technologies and mobile devices has been reported [15–17]. The Apple Watch used in this study has been shown to accurately obtain sleep duration and stages, compared with polysomnography testing [22]. The results of this study need validation with a larger sample size and under various conditions, including comparing low-dose pill users with non-pill users; however, this study suggests that wearable devices can accurately acquire data on sleep–wake rhythms in healthy women.

In this study, a strong negative correlation was found between the sleep midpoint and QP values in healthy women. QP values are statistics calculated using chi-square periodograms and are often used to indicate the robustness of rhythms [23]. The results indicate that the later the sleep midpoint, the greater the variation in the sleep–wake rhythm, suggesting that healthy women with late sleeping habits have a disturbed sleep–wake rhythm. Additionally, a positive correlation was found between the sleep midpoint and the PSQIG, although the difference was not significant. The PSQIG indicates self-perceived sleep habits over the past month, with higher scores indicating poorer sleep quality. Thus, the results suggest that the later the sleep midpoint, the worse the self-perceived quality of sleep. Combined with the correlation results with QP values, the delay in the sleep midpoint is presumed to indicate poor subjective and objective sleep quality. Furthermore, a positive correlation was found between SJL, as indicated by the MCTQ score, and the length of the menstrual cycle. This indicates that the larger the SJL, the longer the menstrual cycle lasts. SJL indicates a discrepancy between sleeping on weekdays with social constraints (such as work, school, and chores) and sleeping on holidays without constraints, consistent with the biological clock [24]. This finding suggests that irregular habits disrupt the variations in female hormones and prolong the menstrual cycle. However, no significant difference was found in the length of the menstrual cycle between groups with large (≥ 1 h) and small (< 1 h) SJLs in female university students [25]. Since the average SJL of the participants in the present study was 0.77 h, a relatively small change in SJL may correlate with the length of the menstrual cycle.

BBT measurements showed fluctuations with the stage of the menstrual cycle. The BBT in women usually has low-temperature and high-temperature phases during the menstrual cycle. During the follicular phase, approximately 2 weeks after the onset of menstruation, estrogen secretion is active, resulting in a low-temperature phase; during the luteal phase following ovulation, progesterone secretion is active, resulting in a high-temperature phase [26]. In the present study, the relative body temperature was higher in the luteal phase after ovulation than in the menstrual and follicular phases. However, BBT varied widely among individuals, and no distinguishable variation could be used to identify the ovulation day. Predictions of the menstrual cycle based on BBT fluctuations are inaccurate [26–28]. These findings indicate that accurately identifying menstrual cycle and ovulation dates using only BBT is challenging. However, we found that the amplitude of BBT positively correlated with the length of the menstrual cycle and SJL. The results of a large-scale cohort study in Japan did not demonstrate a strong correlation between the amplitude of BBT and the length of the menstrual cycle [29]. The discrepancy is considered to be due to this study dividing the menstrual cycle into five stages, allowing for more detailed analysis. Additionally, since there have been no results to date investigating the SJL and amplitude of BBT, future large-scale, detailed studies may clarify the correlation between the amplitude of BBT, the length of the menstrual cycle, and the amount of SJL. These results may be useful for the technology predicting the day of ovulation.

Analysis of the variations in sleep duration, sleep midpoint, and QP values for each menstrual cycle stage showed no variation in sleep duration; however, the sleep midpoint and QP values showed menstrual variation. Based on the menstrual phase, the sleep midpoint was advanced during follicular phase I, delayed during follicular phase II, advanced during luteal phase I, and delayed during luteal phase II. The QP values in follicular phases I and II were significantly higher than those in the menstrual phase and luteal phases I and II. In rodents, the phase of locomotor activity rhythm is advanced, and activity increases on the night of the proestrus [5, 6]. This change is caused by estrogen [7, 8] and alleviated by progesterone [8]. However, rodents do not have a luteal phase, and so the model does not perfectly match the hormonal fluctuations of the human menstrual cycle. The increase in QP values during the follicular phase, when estrogen is relatively high, may be due to increased activity due to estrogen action. Since decreased estrogen causes insomnia during menopause [30], increased estrogen in the follicular phase may make the sleep–wake rhythm more robust. In addition to ovarian steroid hormones, anterior pituitary hormones such as LH, follicle-stimulating hormone, and prolactin also fluctuate with the menstrual cycle. The prolactin level is related to sleep duration and excessive daytime sleepiness [31, 32], so it will be necessary to investigate the relationship between the plasma level of each hormone and the sleep–wake rhythm in detail.

## Conclusions

The present study enabled us to observe variations in the robustness of sleep–wake rhythms associated with different stages of the menstrual cycle through objective and long-term evaluation of sleep–wake rhythms in healthy women. Although increasing the number of participants and verifying the results of this study under various conditions (e.g., medication, medical history, age, race, and region) is necessary, accurately predicting the day of ovulation by combining the acquisition of sleep–wake rhythms using wearable devices and basal body temperature is possible.

## Data Availability

All data for data analysis are available at Mendeley data (doi: 10.17632/76gmpt3jfn.1).
